# Knowledge and attitudes of medical students about clinical aspects of congenital cytomegalovirus infection in newborns: A nationwide cross-sectional study in Greece

**DOI:** 10.3389/fmed.2023.1256704

**Published:** 2023-11-16

**Authors:** Sofia Benou, Eleftheria Hatzidaki, Anna Kalaintzi, Ioanna Grivea, Maria Baltogianni, Vasileios Giapros, Agathi Thomaidou, Kosmas Sarafidis, Sofia Tsichla, Xenophon Sinopidis, Eleni Papachatzi, Aggeliki Karatza, Maria Lagadinou, Theodore Dassios, Gabriel Dimitriou, Vassiliki Papaevangelou, Despoina Gkentzi

**Affiliations:** ^1^Department of Paediatrics, Patras, Greece; ^2^Department of Neonatology/ NICU, School of Medicine, University of Crete, Heraklion, Greece; ^3^Department of Paediatrics, Faculty of Medicine, School of Health Sciences, University of Thessaly, Larissa, Greece; ^4^Neonatal Intensive Care Unit, University of Ioannina, Faculty of Medicine, Ioannina, Greece; ^5^Department of Neonatology, School of Medicine, Aristotle University of Thessaloniki, Hippokrateion General Hospital, Thessaloniki, Greece; ^6^Department of Internal Medicine, Patras, Greece; ^7^Third Department of Paediatrics, National and Kapodistrian University of Athens, School of Medicine, University General Hospital ATTIKON, Chaidari, Athens, Greece

**Keywords:** congenital CMV, medical students, knowledge, attitudes, practices

## Abstract

**Introduction:**

Cytomegalovirus (CMV) is the most frequent cause of congenital infection worldwide causing severe morbidity in newborns, infants, and children. Despite the clinical importance of congenital CMV (cCMV) infection, studies conducted so far indicate that there is limited awareness in the medical community in the field. The aim of this study was to assess Greek medical students’ knowledge on cCMV infection.

**Methods:**

We performed a questionnaire-based nationwide cross-sectional study. A convenience sample of medical students from seven medical schools was enrolled.

**Results:**

Of the 562 respondents, 54,8% considered themselves undereducated on cCMV infection. However, almost half of the participants could correctly recognize some basic principles of cCMV infection including ways of transmission, diagnosis and treatment, while there were aspects of cCMV infection with knowledge deficit. The year of study had a positive impact on the level of knowledge with students of higher years of study being of more sufficient education on the specific topic.

**Conclusion:**

Overall, our study indicates a discrepancy between self-reported awareness and the level of knowledge among medical students in Greece. Further educational opportunities about cCMV should be offered, particularly in areas of the curriculum involving the care of women and children. Establishing medical students’ solid background on the disease burden and educating them about preventative strategies for at-risk populations, should be the main pillars of such efforts in order to promote confidence in managing these cases in their future professional careers.

## 1 Introduction

Cytomegalovirus (CMV) is the most common cause of congenital infection worldwide, affecting 0.2 to 2% of all live births and the main non-genetic cause of congenital sensorineural hearing loss and neurodevelopmental abnormalities in developed countries (1, 2). Among infected newborns, approximately 10% present symptomatic infection at birth (1). Clinical manifestations include hepatosplenomegaly, jaundice, petechial/purpuric rash, auditory and visual impairment, and neurologic abnormalities such as microcephaly (3). Approximately 40–60% of symptomatic newborns will develop a permanent disability, while among newborns with asymptomatic congenital CMV (cCMV), an estimated 10–15% will develop long-term sequelae (4, 5). Long-term effects of congenital CMV include sensorineural hearing loss (SNHL), cognitive impairment, retinitis, and/or cerebral palsy (3, 5).

The highest risk of transmission of CMV to the fetus occurs during a primary CMV infection of the mother; however, in seropositive mothers, reactivation of a latent virus or reinfection with a new CMV strain can also affect the fetus, causing cCMV disease with or without sequelae in the newborn and child (6). Interestingly, in populations with high seroprevalence, more infants with cCMV born to mothers who have had previous CMV infection are expected to be born compared to women with primary seroconversion in pregnancy (7, 8). Vertical transmission after maternal primary CMV infection increases with advancing pregnancy. However, the likelihood of fetal complications is higher when CMV infection happens early in pregnancy, during the first trimester (9). Spreading of CMV occurs through close contact with infected body fluids and children between 1 to 2 years are the most important source of infection for women of reproductive age (1). Prenatal hygiene counseling has been shown to decrease maternal CMV infection and therefore the risk of transmitting CMV to the fetus (10).

It is difficult to ascertain the prevalence of the disease burden because most children are asymptomatic at birth and newborn screening is not universally applied. However, the economic burden of the disease and its sequelae is estimated to be similar to that for congenital rubella before the introduction of vaccination (11). The healthcare resource utilization by infants with cCMV disease is on average seven times greater than healthy infants during the first year of life (12).

Despite all the above, low awareness about cCMV infection among both pregnant women and healthcare professionals has been reported in the literature (5). A systematic review on the healthcare professionals’ level of awareness about cCMV, concluded that healthcare specialists seem to underestimate the prevalence of cCMV infection leading to suboptimal patient information with an increased risk of maternal infection during pregnancy as well as to misdiagnosis or delay in the management of typically symptomatic children with cCMV infection (5). As a result, education of healthcare professionals on cCMV infection and preventive measures for maternal CMV infection is of paramount importance.

Interestingly, in a study conducted in the United States in 2014, which assessed medical students’ knowledge on cCMV, an expected and significant difference was noticed between first and last year medical students, with a sharp increase in awareness of infection’s transmission routes and newborn’s clinical manifestations (5, 13).

The aims of our study were to (a) assess the knowledge and attitudes of medical students in Greek Universities towards cCMV and (b) investigate their self-perceived behaviors about cCMV in order to identify knowledge gaps that highlight opportunities for medical education. The results of this study may assist the development of targeted training activities which might improve healthcare professionals’ ability and skills to identify and manage cCMV infection.

## 2 Materials and methods

### 2.1 Study design, participants, and questionnaire

This is a nationwide cross-sectional knowledge, attitudes and practices (KAP) study conducted in Greece. The questionnaire-based survey was addressed to medical students in all seven medical schools of the Greek public Universities (of note no private medical schools exist in the country). The questionnaire was distributed from September to December 2022. Medical students during their rotation on patient wards were asked to participate. The duration of studying Medicine in Greece is six years, including three years of rotation on the patient wards of all subspecialties (4th, 5th, and 6th year) and all medical students studying in Greek Universities are considered of similar educational level as there are national entrance exams in order to achieve in medical school. Until their 4^th^ year of studies, all medical students in Greek Universities have finished their theoretical education on both preclinical and clinical subjects. The questionnaire was written in Greek, it was distributed on paper and the completed questionnaires was recollected anonymously by the researcher. All medical students being on hospital for their rotation on patient wards were applicable for participation.

A structured questionnaire was developed by two members of the research team (SB, DG) based on previous studies (5, 13, 14). Pilot testing was conducted prior to study initiation and appropriate adjustments were performed. It included questions on cCMV infection, symptoms and signs of CMV infection in healthy adults and newborns, transmission routes, treatment options and existence of a licensed vaccine. The questionnaire was related to the clinical aspect of cCMV in children and the selection of questions related to laboratory diagnostics was guided under the spectrum assumed to be more relative to the clinical aspect of the infection. The suggested answers were based on the literature, and some were incorrect (i.e., false symptoms). Multiple answers were an acceptable option and there were not open-ended questions. The questionnaire consisted of four parts according to KAP studies guidelines.^15^^,^^16^ Part A included six questions on demographics (gender, age, university, year of study, parenthood, and future medical specialty). Part B included 13 questions regarding knowledge about cCMV. Part C included five questions regarding the education they had received from their Faculty’s curriculum or elsewhere and it evaluated their opinions about cCMV infection. In this part, a total of 27 clinical conditions or infectious diseases were given on a 5-point Likert scale (“extremely well,” “well,” “moderately,” “little,” or “not at all”; or “very much,” “much,” “moderately,” “little,” or “not at all”). The scope was the self-assessment of the participants’ level of familiarity. Part D included five questions regarding practices of cCMV management (Supplemental Material 1).

The study was conducted according to the guidelines of the Declaration of Helsinki and approved by the Research Ethics Committee of the University of Patras, Greece (ID: 8383/25.09.2021). An overview of the study objectives was provided to the potential participants, and consent was obtained.

### 2.2 Statistical analysis

Descriptive statistics were initially conducted to summarize the data on demographic characteristics and knowledge, attitudes, and practices regarding cCMV. Statistical analysis was performed using the SPSS Statistical Software Package (IBM SPSS Statistics, version.26, Armonk, NY, United States). Since all questions were dichotomous or categorical, the Pearson Chi-squared test was used to determine any significant associations between the demographic characteristics and a student’s knowledge and attitude. In all cases, the level of statistical significance was set to *p* < 0.05. During the statistical analysis, the answers “moderately,” “little,” and “not at all” were grouped as “below average,” whereas the rest were grouped as “above average.”

## 3 Results

### 3.1 Demographic characteristics of medical students

A total of 600 questionnaires were distributed to 4th, 5th and 6th grade medical students. Among them, 562 students completed the survey and were included in the final analysis (response rate: 94%). Most were females (56%), studying during the final year of medical training (39%). The mean age of participants was 23.6 years old (Supplementary Figure S1.) and only 2% of them were parents. Demographic characteristics of the participants are presented in Table 1.

**Table 1:** Demographic characteristics of the study population (*n* = 562).

	Number (*N*)	Percentage %
Total number of students	562	
**Gender**		
Male	233	41.5
Female	315	56.0
Other	14	2.5
**Year of studies**		
4^th^	147	26.2
5^th^	170	30.3
6^th^	219	39.0
Pending graduation	25	4.4
**University**		
National and Kapodistrian University of Athens	17	3.0
Aristotle University of Thessaloniki	55	9.8
University of Patras	96	17.1
University of Ioannina	56	10.0
University of Thessaly	107	19.0
University of Crete	225	40.0
Democritus University of Thrace	6	1.1
**Parent of at least one child**		
Yes	11	2.0
No	545	98.0
**Future medical speciality intention**		
Family Medicine	4	0.7
General Surgery	38	6.8
Internal Medicine	42	7.5
Pediatrics	49	8.8
Psychiatry	26	4.7
Obstetrics- Gynecology	19	3.4
Other internal medicine subspeciality	94	16.8
Other surgical subspeciality	103	18.5
Research in Medicine	10	1.8
Undecided	143	25.6
More than 2 options	30	5.4

### 3.2 Medical students’ knowledge of cCMV infection

Among participants, only 8/562 (1.4%) answered not being aware of cCMV infection. Descriptive statistics on general knowledge related to cCMV are presented in Table 2. The majority, 61% could identify the latency of CMV and the possibility of reactivation in periods of impaired immunity (85.8%). Almost half of the participants (52.4%) correctly mentioned cCMV infection as the most frequent cause of congenital infection. Conversely, 380 of them (69.6%) could recognize cCMV infection as the main nongenetic cause of congenital sensorineural hearing loss and neurodevelopmental abnormalities in children. Most students could correctly identify those with the highest risk for severe sequalae after CMV infection. Fetuses (70.8%), pregnant women (83.3%), immunosuppressed or HIV patients (81.5%) and solid organ transplant recipient patients (65.7%) were correctly recognized as at-risk populations.

**Table 2:** General knowledge of the study population regarding cCMV infection (*n* = 562).

	Number (%)			
	True	False	Do not know	Total
CMV is a double-stranded DNA virus and is a member of the herpesviruses.	414 (75.4)	86 (15.7)	49 (8.9)	549 (97.3)
CMV reactivation may occur during prolonged periods of impaired immunity	471 (85.8)	51 (9.3)	27 (4.9)	549 (97.3)
CMV causes latent infection in the host after primary infection	328 (61.0)	122 (22.7)	88 (16.4)	538 (95.7)
CMV is the most frequent cause of congenital infection	285 (52.4)	154 (28.3)	105 (19.3)	544 (96.8)
cCMV infection is the main nongenetic cause of congenital sensorineural hearing loss and neurodevelopmental abnormalities in children	380 (69.6)	64 (11.7)	102 (18.7)	546 (97.1)
Among which of the following population should CMV infection be prevented? (multiple answers)				
Fetus				398 (70.8)
Pregnant women				471 (83.8)
Immunosuppressed patients				458 (81.5)
Immunocompetent patient				89 (15.8)
Children				45 (8.0)
Solid organ transplant patients				369 (65.7)
Diagnosis of cCMV infection (multiple answers)				
CMV DNA in the amniotic fluid (prenatal diagnosis)				269 (48.2)
Maternal serology (IgM+, IgG+) (prenatal diagnosis)				340 (60.9)
CMV PCR in saliva or urine collected within 3 weeks of life (neonatal diagnosis)				309 (55.4)
There are available antiviral agents for the treatment of cCMV infection	325 (59.3)	83 (15.1)	140 (15.5)	548 (97.5)
Treatment options				
Ganciclovir or Valganciclovir				228 (40.5)
Other				243 (50.1)
There is a licensed vaccine for prevention of cCMV infection	49 (9.1)	343 (63.4)	148 (27.5)	540 (96)

Regarding diagnosis of cCMV infection, almost half of the participants recognized both prenatal diagnosis by detecting CMV DNA in the amniotic fluid and diagnosis in the newborn by detecting CMV DNA in saliva or urine collected within the first 21 days of life (48.2 and 55.4% respectively). Moreover, half of the participants were aware of the available treatment for cCMV infection and could recognize the available antiviral agents (40.5%). When asked whether a licensed vaccine is available for CMV infection, 343 participating students correctly answered about the lack of an approved vaccine and 148 (27.5%) answered “Do not know.” (Table 2).

Table 3 presents the chi-square distribution analysis of medical students’ awareness of the ways of transmission of cCMV associated with their year of study. Significant differences were observed between 4^th^- year medical students (MSΥ4) and 6^th^-year medical students (MSΥ6). The earlier year of study was associated with less knowledge about CMV transmission routes. The 5^th^-year medical students (MSΥ5) selected a more significant percentage (76.5%) saliva or kiss as a way of transmission of CMV than MSΥ4 (60.5%). A statistically significant difference was noticed between awareness of blood transfusions, solid organ transplantations and sexual contact as ways of transmission between MSΥ4 and MSΥ5-MSΥ6, with the last of them presenting a higher awareness. Moreover, MSY4 presented lower rates of awareness about contact with wet diapers (9.5%) and breastfeeding (38.8%) as ways of CMV transmission in comparison to MSΥ5- MSΥ6. However, the total awareness about contact with urine or stool was low, with only 16% being aware of this transmission route. Interestingly, less than the half of the participants (42%) were aware of coughing or sneezing as a way of transmitting CMV with no significant difference among the year of studies.

**Table 3:** Comparison between medical student classes and knowledge and awareness of routes of CMV transmission.

	Total	4^th^ Year	5^th^ Year	6^th^ Year	p.value
	*N* (%)	*N* (%)	*N* (%)	*N* (%)	*x*^2^-test
Saliva/ Kissing					
Yes	368 (68.7)	89 (60.5)	130 (76.5)	149 (68.0)	**0.009**
No	168 (31.3)	58 (39.5)	40 (23.5)	70 (32.0)	
Organ or marrow transplant					
Yes	290 (54.1)	54 (36.7)	106 (62.4)	130 (59.4)	**0.000**
No	246 (45.9)	93 (63.3)	64 (37.6)	89 (40.6)	
Contact with cat litter					
Yes	25 (4.7)	3 (2.0)	14 (8.2)	8 (3.7)	**0.022**
No	511 (95.3)	144 (98.0)	156 (91.8)	211 (96.3)	
Coughing/ Sneezing					
Yes	225 (42.0)	63 (42.9)	62 (36.5)	100 (45.7)	0.184
No	311 (58.0)	84 (57.1)	108 (63.5)	119 (54.3)	
Blood transfusions					
Yes	311 (58.1)	73 (49.7)	101 (59.4)	137 (62.8)	**0.040**
No	224 (41.9)	74 (50.3)	69 (40.6)	81 (37.2)	
Contact with birds					
Yes	7 (1.3)	2 (1.4)	3 (1.8)	2 (0.9)	-
No	529 (98.7)	145 (98.6)	167 (98.2)	217 (99.1)	
Contact with stool or urine (wet diapers)					
Yes	90 (16.8)	14 (9.5)	39 (22.9)	37 (16.9)	**0.006**
No	446 (83.2)	133 (90.5)	131 (77.1)	182 (83.1)	
Sharing eating utensils					
Yes	142 (26.5)	36 (24.5)	39 (22.9)	67 (30.6)	0.193
No	394 (73.5)	111 (75.5)	131 (77.1)	152 (69.4)	
Eating undercooked meat					
Yes	14 (2.6)	2 (1.4)	6 (3.5)	6 (2.7)	-
No	522 (97.4)	145 (98.6)	164 (96.5)	213 (97.3)	
Contact with rodents					
Yes	8 (1.5)	3 (2.0)	4 (2.4)	1 (0.5)	-
No	528 (98.5)	144 (98.0)	166 (97.6)	218 (99.5)	
Sexual intimacy					
Yes	218 (40.7)	39 (26.5)	92 (54.1)	87 (39.7)	**0.000**
No	318 (59.3)	108 (73.5)	78 (49.5)	132 (60.3)	
Breastfeeding					
Yes	266 (49.6)	57 (38.8)	99 (58.2)	110 (50.2)	**0.002**
No	270 (50.4)	90 (61.2)	71 (41.8)	109 (49.8)	
Do not know					
Yes	5 (0.9)	2 (1.4)	1 (0.6)	2 (0.9)	-
No	531 (99.1)	145 (98.6)	169 (99.4)	217 (99.1)	

A x^2^ test was used to determine the strength of correlation between the measured variables.

The level of statistical significance is set at 5%.

Regarding clinical manifestations, most medical students could recognize the clinical impact among symptomatic infants and children (Table 4). A correlation between knowledge of clinical manifestations and the year of study was also assessed and a similar pattern was observed regarding knowledge on cCMV manifestations. Level of awareness differed significantly between MSΥ5-MSΥ6 and MSΥ4. Indeed, the former presented higher awareness about intrauterine growth restriction (IUGR), chorioretinitis, sensorineural hearing loss (SNHL) and hepatosplenomegaly as some of the clinical manifestations of symptomatic cCMV infection in comparison to the latter. Remarkably, almost half of the participants knew that cCMV can cause brain abnormalities, microcephaly and cognitive impairment without difference among the year of studies.

**Table 4:** Comparison between medical student’s year and knowledge about signs, symptoms and sequalae associated with cCMV infection.

	Total	4th Year	5th Year	6th Year	*p*.value
	*N* (%)	*N* (%)	*N* (%)	*N* (%)	*x*^2^-test
**Intrauterine growth restriction (IUGR)**					
Yes	332 (61.9)	85 (57.8)	93 (54.7)	154 (70.3)	**0.003**
No	204 (38.1)	62 (42.2)	77 (45.3)	65 (29.7)	
**Chorioretinitis**					
Yes	267 (49.8)	54 (36.7)	80 (47.1)	133 (60.7)	**0.000**
No	269 (50.2)	93 (63.3)	90 (52.9)	86 (39.3)	
**Sensorineural hearing loss (SNHL)**					
Yes	447 (83.4)	114 (77.6)	132 (77.6)	201 (91.8)	**0.000**
No	89 (16.6)	33 (22.4)	38 (22.4)	18 (8.2)	
**Brain abnormalities**					
Yes	273 (50.9)	70 (47.6)	87 (51.2)	116 (53.0)	0.603
No	263 (49.1)	77 (52.4)	83 (48.8)	103 (47)	
**Microcephaly**					
Yes	229 (42.7)	62 (42.2)	67 (39.4)	100 (45.7)	0.460
No	307 (57.3)	85 (57.8)	103 (60.6)	119 (54.3)	
**Congenital heart disease**					
Yes	91 (17.0)	29 (19.7)	32 (18.8)	30 (13.7)	0.238
No	445 (83.0)	118 (80.3)	138 (81.2)	189 (86.3)	
**Seizures**					
Yes	142 (26.5)	40 (27.2)	37 (21.8)	65 (29.7)	0.209
No	394 (73.5)	107 (72.8)	133 (78.2)	154 (70.3)	
**Arthritis**					
Yes	24 (4.5)	7 (4.8)	8 (4.7)	9 (4.1)	0.943
No	512 (95.5)	140 (95.2)	162 (95.3)	210 (95.9)	
**Cognitive impairment**					
Yes	332 (61.9)	83 (56.5)	101 (59.4)	148 (67.6)	0.071
No	204 (38.1)	64 (43.5)	69 (40.6)	71 (32.4)	
**Hepatosplenomegaly**					
Yes	237 (44.2)	52 (35.4)	77 (45.3)	108 (49.3)	**0.029**
No	299 (55.8)	95 (64.6)	93 (54.7)	111 (50.7)	
**Neonatal jaundice**					
Yes	144 (26.9)	40 (27.2)	44 (25.9)	60 (27.4)	0.940
No	392 (73.1)	107 (72.8)	126 (74.1)	159 (72.6)	
**Blood stool**					
Yes	25 (4.7)	5 (3.4)	10 (5.9)	10 (4.6)	0.577
No	511 (95.3)	142 (96.6)	160 (94.1)	209 (95.4)	
**Do not know**					
Yes	6 (1.1)	2 (1.4)	3 (1.8)	1 (0.5)	-
No	529 (98.9)	145 (98.6)	167 (98.2)	217 (99.5)	

A x^2^ test was used to determine the strength of correlation between the measured variables.

The level of statistical significance is set at 5%.

### 3.3 Medical students’ attitudes on education about cCMV infection

**Figure 1:**
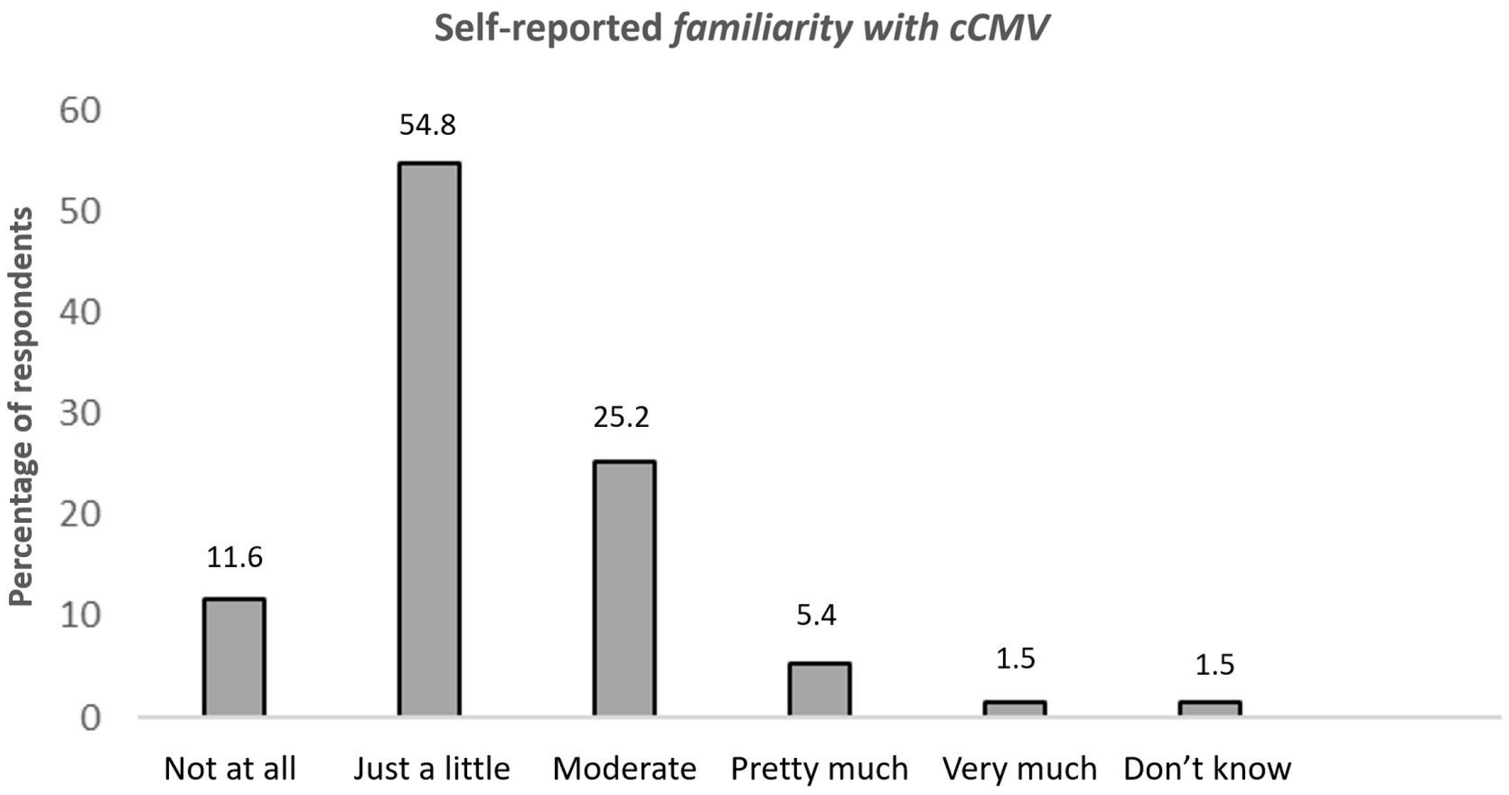
Self-reported familiarity with cCMV infection. cCMV: congenital cytomegalovirus.

Regarding the students’ attitude on cCMV education, the self-estimated level of awareness about cCMV was low (Figure 1). Among the respondents, 54.8% stated that their familiarity with cCMV infection was low (Figure 1), while only 38 (6.9%) of them self-reported being very familiar with cCMV. When asked about their education, most of the students (61.2%) mentioned feeling unsatisfied about the cCMV education provided by their institution (Table 5). Among the participants, 63.3% admitted having learned about cCMV at some point during medical school while 10.2% mentioned the internet as a source of education. Some participants (22.5%) admitted having received education by both University and internet (Table 5).

**Table 5:** Education on cCMV infection.

Satisfaction about curriculum-based education on cCMV infection					
	Not at all	A little	Moderate	Pretty much	Very much
**N (%)**	34 (6.3)	173 (31.8)	160 (29.4)	154 (28.3)	23 (4.2)
Source of education about CMV					
	**University**	**Internet**	**Television**	**Personal experience**	**University and Internet**
**N (%)**	337 (63.6%)	54 (10.2)	1 (0.2)	13 (2.5)	119 (22.5)

Participants were also asked to self-report their familiarity with other common clinical conditions and infectious diseases. Severe acute respiratory coronavirus 2 (SARS-CoV2), myocardial infraction and breast cancer were characterized as the most familiar clinical conditions by most of the participants (Table 6). Regarding other congenital infections, self-reported awareness about cCMV (6.9%) was lower that congenital toxoplasmosis (17.9%) and congenital rubella syndrome (11.7%). A two-tailed Pearson test was used to test the statistical significance between these clinical conditions and cCMV level of self-reported familiarity. All the investigated clinical conditions presented a linear correlation with cCMV infection (*p* < 0.01), meaning that as the level of awareness was increasing for these conditions, it was also increasing for cCMV infection.

**Table 6:** Self-reported familiarity with common clinical conditions and infectious diseases in correlation with self-reported familiarity with CMV.

	Self-reported familiarity*	CMV	
	*N* (%)	Correlation coefficient (r)	*p*.value
CMV	38 (6.9)	**-**	**-**
SARS-CoV2	340 (61.9)	0.192**	**0.000**
Congenital toxoplasmosis	98 (17.9)	0.353**	**0.000**
Congenital rubella syndrome	64 (11.7)	0.567**	**0.000**
Upper respiratory tract infections	230 (42.7)	0.227**	**0.000**
Myocardial infraction	324 (59.8)	0.146**	**0.001**
Colon cancer	284 (52)	0.134**	**0.002**
Breast cancer	301 (54.6)	0.190**	**0.000**
Down syndrome	226 (41.1)	0.216**	**0.000**
Respiratory distress syndrome	26 (5.5)	0.391**	**0.000**

* Self-reported familiarity is characterized by the answers “pretty much” and “very much” familiar with each one of the clinical conditions.

** Correlation is significant at the 0,01 level (2-tailed).

The level of statistical significance is set at 5%.

### 3.4 Medical students’ attitudes and practices on cCMV infection

Attitudes towards disease burden were relatively homogenous among medical students (Table 7). Almost all of them admitted that the disease burden is significant for the family (95.4%) and that symptomatic cCMV infection can affect infant’s quality of life (97.6%). In terms of clinical practice, almost all students (95.9%) agreed that a licensed vaccine could contribute critically to prevention of cCMV infection. Similarly, almost 100% agreed on the critical role of prevention counseling to pregnant women in reducing the prevalence of the maternal infection.

**Table 7:** Comparison of cCMV attitudes and practices to demographic characteristics of population.

	Total	Disagree/Strongly disagree	Agree/ Strongly agree	*p*.value
	N (%)	N (%)	N (%)	
A licensed vaccine could contribute critically to prevention of cCMV infection				
**Gender**				
Female	254 (57.6)	10 (3.9)	244 (96.1)	0.858*
Male	187 (42.4)	8 (4.3)	179 (95.7)	
**Year of studies**				
5^th^	131 (42.0)	4 (3.1)	127 (96.9)	0.568**
6^th^	181 (58.0)	5 (2.8)	176 (97.2)	
The disease burden of cCMV infection for the family is significant				
**Gender**				
Female	238 (57.8)	11 (4.6)	227 (95.4)	0.991*
Male	174 (42.2)	8 (4.6)	166 (95.4)	
**Year of studies**				
5^th^	122 (40.9)	7 (5.7)	115 (94.3)	0.035**
6^th^	176 (59.1)	2 (1.1)	174 (98.9)	
Clinical manifestations of cCMV infection affect the quality of life of the infant				
**Gender**				
Female	271 (57.7)	7 (2.6)	264 (97.4)	0.766**
Male	199 (42.3)	4 (2.0)	195 (98.0)	
**Year of studies**				
5^th^	138 (41.6)	3 (2.2)	135 (97.8)	0.311**
6^th^	194 (58.4)	1 (0.5)	193 (99.5)	
CMV prevention counseling should be provided to all pregnant women				
**Gender**				
Female	288 (57.7)	2 (0.7)	286 (99.3)	0.247**
Male	211 (42.3)	4 (1.9)	207 (98.1)	
**Year of studies**				
5^th^	149 (42.6)	3 (2.0)	146 (98.0)	0.654**
6^th^	201 (57.4)	2 (1.0)	199 (99.0)	

*x^2^-test, **Fisher’s exact test.

The level of statistical significance is set at 5%.

The multivariable linear regression analysis showed no significant statistical difference among cCMV practices and demographic characteristics of participants, indicating that all students unanimously wish for preventive measures and counseling regarding cCMV infection (Table 7).

## 4 Discussion

To the best of our knowledge, this is the first study performed on the knowledge and attitudes of medical students in Greece regarding cCMV infection. The response rate was 94% and it included medical students at a nationwide scale. Our study differs from previous studies in the field because it was conducted according to KAP studies guidelines (15, 16). A KAP survey is a quantitative method of data collection in order to quantify and measure a phenomenon. This type of survey is conducted through questionnaires intended to identify critical knowledge, social skills, and behaviors commonly shared by a population or target group about particular issues. Previous studies in the field mainly evaluating the level of awareness or knowledge among participants about cCMV and similar studies in the field of congenital infections were lacking (17, 18). The qualitative insight provided by this study shows that the rates of awareness amongst medical students are moderate with significant knowledge gaps. Almost half of the participants were able to recognize the basic principles of the pathophysiology of the disease as well as diagnostic methods and available treatment. These results are better than the levels of awareness among students of other healthcare professions such as midwifery students and nurses (19).

Regarding ways of transmission, the level of knowledge varied from low to moderate with the majority of medical students being aware of transmission through saliva, organ or blood marrow, blood transfusion, sexual contact, and breastfeeding. However, few students were aware of the transmission of CMV through contact with stools or urine and sharing eating utensils. Interestingly, only 16,9% of upper-level students recognized that cCMV could be transmitted by sharing food or drink. Moreover, almost half of the participants could recognize the most common clinical manifestations of symptomatic cCMV infection. These levels are similar to the levels of knowledge on clinical manifestations previously reported among the general population (20). The latter highlights the need to strengthen the medical curriculum to adequately train future physicians. A statistically significant difference was observed between MSY5-MSY6 and MSY4 level of awareness. These results are quite optimistic for Greek universities because they underline the educational impact of the curriculum during the years of undergraduate training. Similar findings were observed in a study conducted in a single institution in 2014 (13).

Despite the moderate level of knowledge of medical students, the self-reported awareness about cCMV infection presented to be low with 54.8% feeling undereducated. According to our findings, the education provided by the medical curriculum was considered insufficient by the students themselves because only 32.5% were self-characterized as fully educated. However, the self-reported awareness of other common clinical conditions had a positive correlation with cCMV infection (*p* < 0,05), meaning that as the level of awareness was increasing for these conditions, it was also increasing for cCMV infection. Notably, most students were receiving education for cCMV delivered only by the university curriculum.

Other studies have documented a worldwide lack of knowledge among medical professionals in a disproportionate way compared to the high incidence of the disease. Surveys of various healthcare professionals worldwide also indicate a gap in both awareness and knowledge of cCMV. Studies conducted in Australia and France among obstetricians/gynecologists, general practitioners and midwives revealed lack of confidence and knowledge about cCMV infection (17, 21). Regarding otologists, a study concluded in the US also indicated several knowledge gaps and underutilization of cCMV testing by physicians who frequently encounter paediatric hearing loss (22).

However, it is essential for physicians to be aware of the disease burden of the infection and the routes of CMV transmission in order to properly counsel their patients and families. In the absence of a licensed effective vaccine, the best primary prevention strategy for CMV infection in pregnancy is education on hygiene precautions to reduce the risk of maternal infection (23). This highlights that there is a great need for a more structured systematic approach to the training of future healthcare professionals in this field. The opportunities for better education should begin early in their career preferably at an undergraduate level, while undoubtedly healthcare providers should strive for continuous professional development in the field (5).

A more structured and thorough education on cCMV would also help medical students feel more confident in managing cases of cCMV but also educate healthcare professionals in CMV prevention. The risk of acquiring CMV during pregnancy can be significantly decreased if allied healthcare professionals adopt specific behavioral changes including hand washing after contact with wet diapers (24). Interestingly, in terms of clinical practice, almost all medical students highlighted the importance of primary prevention of this congenital infection. In addition, medical students should be aware that mononucleosis symptoms during pregnancy might be indicative of CMV infection. Hence, they will be able to early identify and manage the consequences of the infection the soonest possible (25).

Our study adds to the limited body of the literature in the field and raises further public health considerations. Given the enhanced understanding that we currently have on cCMV infection, (23) medical education should also focus on the area and begin as soon as possible. However, our study as pragmatic one, has several limitations that we have to acknowledge. Firstly, some of the faculties contributed a limited number of participants and there was an unequal number of participants by years of study. Our results, however, could be considered representative of the country, as in Greece all medical students meet specific entry requirements, and they can hence be regarded as an homogenous population. We cannot, however, generalize our findings to other countries, as curriculums differ between countries. Our questions were mainly orientated on the diagnostics and management in neonates. We mainly included clinical oriented questions and we did not cover aspects of serological diagnostics or differences between primary infection and reactivation. Moreover, we did not access actual medical practices relevant to cCMV in this study because medical students do not have unsupervised clinical duties. These aspects could serve as the goal of a future study in the field at a postgraduate level of perinatal care healthcare professionals. In addition, the participation in our study was voluntary. Therefore, students feeling unaware about cCMV may have deliberately decided not to participate, causing some selection bias.

In conclusion, our cross-sectional study indicates that medical students in Greece have moderate awareness of cCMV infection. Despite the low self-reported awareness, almost all students endorsed being willing to learn more about cCMV infection. Further educational opportunities about cCMV should be offered, particularly in subjects of the curriculum involving the care of women and children. Establishing medical students’ solid knowledge background on the disease burden and educating them about preventative strategies for at-risk populations, should be the main pillars of such efforts in order to promote confidence in managing these cases in their future professional careers.

## Data availability statement

The raw data supporting the conclusions of this article will be made available by the authors, without undue reservation, after request.

## Author contributions

SB: Conceptualization, Data curation, Formal analysis, Investigation, Methodology, Project administration, Writing – original draft, Writing – review & editing. EH: Investigation, Writing – review & editing. AnK: Investigation, Writing – review & editing. IG: Writing – review & editing. MB: Investigation, Writing – review & editing. VG: Writing – review & editing. AT: Investigation, Writing – review & editing. KS: Writing – review & editing. ST: Data curation, Writing – review & editing. XS: Writing – review & editing. EP: Writing – review & editing. AgK: Writing – review & editing. ML: Writing – review & editing. TD: Writing – review & editing. GD: Supervision, Writing – review & editing. VP: Supervision, Writing – review & editing. DG: Conceptualization, Methodology, Project administration, Supervision, Writing – review & editing.
