# Supplementary material for: Knowledge and attitudes of medical students about clinical aspects of congenital cytomegalovirus infection in newborns: A nationwide cross-sectional study in Greece
Source: Front Med (Lausanne). 2023 Nov 16;10:1256704. doi: 10.3389/fmed.2023.1256704 (PMC10687632; doi:10.3389/fmed.2023.1256704)
Supplement: Supplementary file 1 [file Data_Sheet_1.docx]

Supplementary Material

**QUESTIONAIRE FOR MEDICAL STUDENTS**

**KNOWLEDGE, ATTITUDES AND PRACTISES ABOUT CONGENITAL CMV INFECTION**

1. **DEMOGRAPHICS**

| **QUESTION** | **RESPONSE OPTIONS** |
| --- | --- |
| **Gender** | Male  Female  Other |
| **Year of studies** | 4^th^  5^th^  6^th^  Pending graduation |
| **University** | National and Kapodistrian University of Athens  Aristotle University of Thessaloniki  University of Patras  University of Ioannina  University of Thessaly  University of Crete  Democritus University of Thrace |
| **Parent of at least one child** | Yes  No |
| **Future medical specialty intention** | Family Medicine  General Surgery  Internal Medicine  Pediatrics  Psychiatry  Obstetrics- Gynecology  Other internal medicine subspeciality  Other surgical subspeciality  Research in Medicine  Undecided |

**B. KNOWLEDGE**

| **QUESTION** | **RESPONSE OPTIONS** |
| --- | --- |
| **Do you know what is cCMV infection?** | **YES**  **NO** |
| **For each one of the following sentences mark as “True” or “False”** |  |
| *CMV is a double-stranded DNA virus and is a member of the herpesviruses.* | TRUE  FALSE  DON’T KNOW |
| *CMV reactivation may occur during prolonged periods of impaired immunity* | TRUE  FALSE  DON’T KNOW |
| *CMV causes latent infection in the host after primary infection* | TRUE  FALSE  DON’T KNOW |
| *cCMV infection is the main nongenetic cause of congenital sensorineural hearing loss and neurodevelopmental abnormalities in children* | TRUE  FALSE  DON’T KNOW |
| *CMV is the most frequent cause of congenital infection* | TRUE  FALSE  DON’T KNOW |
| **Among which of the following population should CMV infection be prevented? (multiple answers)** | Fetus  Pregnant women  Immunosuppressed patients  Immunocompetent patient  Children  Solid organ transplant patients |
| **Diagnosis of cCMV infection (multiple answers)** | - CMV DNA in the amniotic fluid (prenatal diagnosis). - Maternal serology (IgM+, IgG+) (prenatal diagnosis) - Clinical manifestations indicative of cCMV in the neonate - Detection of congenital anatomical anomalies in second trimester ultrasound during pregnancy - CMV PCR in saliva or urine collected within 3 weeks of life (neonatal diagnosis) |
| **Through which one of the following routes can CMV transmitted? (multiple answers)** | - Saliva/ Kissing - Organ or marrow transplant - Contact with cat litter - Coughing/ Sneezing - Blood transfusions - Contact with birds - Contact with stool or urine (wet diapers) - Sharing eating utensils - Eating undercooked meat - Contact with rodents - Sexual intimacy - Breastfeeding - Don’t know |
| **Clinical manifestations of cCMV infection (multiple answers)** | Intrauterine growth restriction (IUGR)  Chorioretinitis  Sensorineural hearing loss (SNHL)  Brain abnormalities  Microcephaly  Congenital heart disease  Seizures  Arthritis  Cognitive impairment  Hepatosplenomegaly  Neonatal jaundice  Blood stool  Don’t know |
| **There are available antiviral agents for the treatment of cCMV infection** | YES  NO  DON’T KNOW |
| **Treatment options for cCMV infection** | Ganciclovir  Valganciclovir  Foscarnet  Ampicillin  Cidofovir  Vitamin A  Doxycyclin |
| **There is a licensed vaccine for prevention of cCMV infection** | YES  NO  DON’T KNOW |

1. **ATTITUDES**

**How familiar do you feel about each one of the following clinical conditions or infectious diseases?**

|  | **NOT AT ALL** | **LITTLE** | **MODERATELY** | **PRETTY MUCH** | **VERY MUCH** | **DON’T KNOW/ DON’T ANSWER** |
| --- | --- | --- | --- | --- | --- | --- |
| SARS-CoV2 |  |  |  |  |  |  |
| Upper respiratory tract infections |  |  |  |  |  |  |
| cCMV infection |  |  |  |  |  |  |
| Hypertension |  |  |  |  |  |  |
| Myocardial infraction |  |  |  |  |  |  |
| HIV infection- AIDS |  |  |  |  |  |  |
| Down syndrome |  |  |  |  |  |  |
| Breast cancer |  |  |  |  |  |  |
| Colon cancer |  |  |  |  |  |  |
| Respiratory distress  syndrome |  |  |  |  |  |  |
| Parvovirus B19 |  |  |  |  |  |  |
| Congenital rubella syndrome |  |  |  |  |  |  |
| *Streptococcus pneumoniae* |  |  |  |  |  |  |
| Rotavirus |  |  |  |  |  |  |
| *Escherichia coli* |  |  |  |  |  |  |
| *Congenital toxoplasmosis* |  |  |  |  |  |  |

**How much do you agree with each one of the following statements?**

|  | **Totally disagree** | **Disagree** | **Neither agree nor disagree** | **Agree** | **Totally agree** | **Don’t know/ Don’t answer** |
| --- | --- | --- | --- | --- | --- | --- |
| **A licensed vaccine could contribute critically to prevention of cCMV infection.** |  |  |  |  |  |  |
| **The disease burden of cCMV infection for the family is significant** |  |  |  |  |  |  |
| **Clinical manifestations of cCMV infection affect the quality of life of the infant** |  |  |  |  |  |  |

| **QUESTION** | **RESPONSE OPTIONS** |
| --- | --- |
| **How much satisfied do you feel about curriculum-based education on cCMV infection** | Not at all  A little  Moderate  Pretty much  Very much |
| **Source of education about cCMV** | University  Internet  Television  Personal experience  Other |

1. **PRACTICES**

|  | **Totally disagree** | **Disagree** | **Neither agree nor disagree** | **Agree** | **Totally agree** | **Don’t know/ Don’t answer** |
| --- | --- | --- | --- | --- | --- | --- |
| **CMV prevention counseling should be provided to all pregnant women** |  |  |  |  |  |  |
| **All pregnant women should be provided serology screening for cCMV infection** |  |  |  |  |  |  |
| **In case of IgM (+) in serology of pregnant women, what would you recommend:**   - Amniocentesis - Premature birth and treatment of the neonate - End of pregnancy - None of the above - Don’t know/ Don’t answer |  |  |  |  |  |  |

**SUPPLEMENTARY FIGURE**


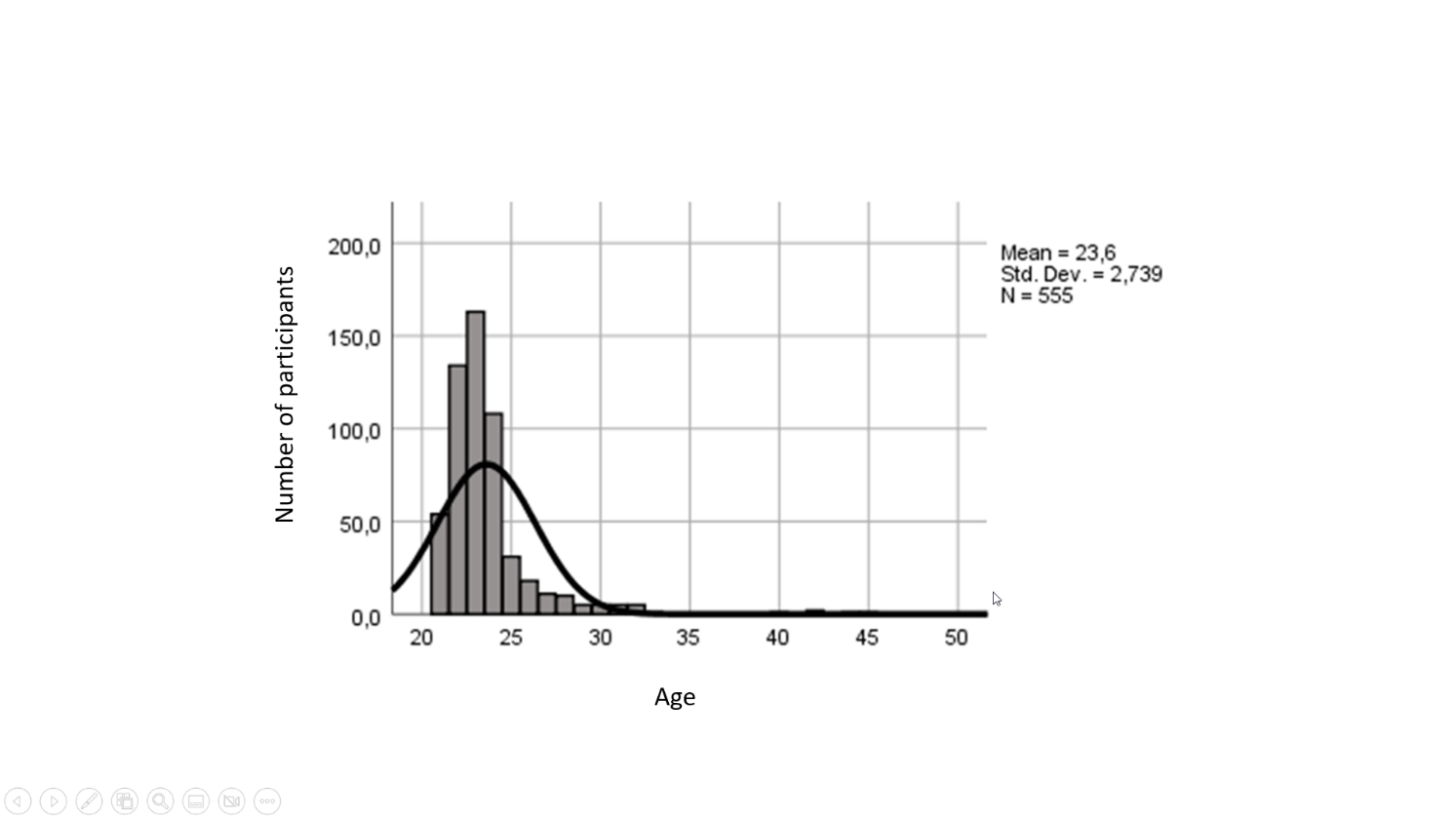


**Supplementary Figure 1. Distribution of respondents’ age**
